# Screening for affective dysregulation in school-aged children: relationship with comprehensive measures of affective dysregulation and related mental disorders

**DOI:** 10.1007/s00787-023-02166-z

**Published:** 2023-02-17

**Authors:** A.-K. Treier, M. Döpfner, U. Ravens-Sieberer, A. Görtz-Dorten, M. Boecker, C. Goldbeck, T. Banaschewski, P.-M. Aggensteiner, C. Hanisch, A. Ritschel, M. Kölch, A. Daunke, V. Roessner, G. Kohls, A. Kaman, for the ADOPT Consortium, Pascal-Maurice Aggensteiner, Tobias Banaschewski, Dorothee Bernheim, Stefanie Bienioschek, Maren Boecker, Daniel Brandeis, Andrea Daunke, Manfred Döpfner, Jörg M. Fegert, Franziska Frenk, Franziska Giller, Claudia Ginsberg, Carolina Goldbeck, Anja Görtz-Dorten, Monja Groh, Charlotte Hanisch, Martin Hellmich, Sarah Hohmann, Nathalie Holz, Christine Igel, Michaela Junghänel, Anna Kaiser, Anne Kaman, Betül Katmer-Amet, Josepha Katzmann, Michael Kölch, Sabina Millenet, Kristina Mücke, Ulrike Ravens-Sieberer, Anne Ritschel, Veit Roessner, Anne Schreiner, Jennifer Schroth, Anne Schüller, Marie-Therese Steiner, Marion Steiner, Anne-Katrin Treier, Anne Uhlmann, Matthias Winkler, Sara Zaplana

**Affiliations:** 1 Department of Child and Adolescent Psychiatry, Psychosomatics and Psychotherapy, Faculty of Medicine and University Hospital Cologne, University of Cologne, Cologne, Germany; 2 School of Child and Adolescent Cognitive Behavior Therapy (AKiP), Faculty of Medicine and University Hospital Cologne, University of Cologne, Pohligstraße 9, 50969 Cologne, Germany; 3 Department of Child and Adolescent Psychiatry, Psychotherapy, and Psychosomatics, University Medical Center Hamburg-Eppendorf, Hamburg, Germany; 4 Department of Child and Adolescent Psychiatry and Psychotherapy, Central Institute of Mental Health, Medical Faculty Mannheim, University of Heidelberg, Mannheim, Germany; 5 Faculty of Human Sciences, University of Cologne, Cologne, Germany; 6 Department of Child and Adolescent Psychiatry, Neurology, Psychosomatics, and Psychotherapy, University Medical Center Rostock, Rostock, Germany; 7 Department of Child and Adolescent Psychiatry/Psychotherapy, University of Ulm, Ulm, Germany; 8 Department of Child and Adolescent Psychiatry and Psychotherapy, TU Dresden, Dresden, Germany; 9 Department of Child and Adolescent Psychiatry, Psychotherapy and Psychosomatics, University Hospital Ruppin-Brandenburg, Neuruppin, Germany; 10 Institute of Medical Statistics and Computational Biology, Faculty of Medicine and University Hospital Cologne, University of Cologne, Cologne, Germany

**Keywords:** Affective dysregulation, Children and adolescents, Screening, Parent report, Validation

## Abstract

Affective dysregulation (AD) is characterized by irritability, severe temper outbursts, anger, and unpredictable mood swings, and is typically classified as a transdiagnostic entity. A reliable and valid measure is needed to adequately identify children at risk of AD. This study sought to validate a parent-rated screening questionnaire, which is part of the comprehensive Diagnostic Tool for Affective Dysregulation in Children (DADYS-Screen), by analyzing relationships with comprehensive measures of AD and related mental disorders in a community sample of children with and without AD. The sample comprised 1114 children aged 8–12 years and their parents. We used clinical, parent, and child ratings for our analyses. Across all raters, the DADYS-Screen showed large correlations with comprehensive measures of AD. As expected, correlations were stronger for measures of externalizing symptoms than for measures of internalizing symptoms. Moreover, we found negative associations with emotion regulation strategies and health-related quality of life. In receiver operating characteristic (ROC) analyses, the DADYS-Screen adequately identified children with AD and provided an optimal cut-off. We conclude that the DADYS-Screen appears to be a reliable and valid measure to identify school-aged children at risk of AD.

**Supplementary Information:**

The online version contains supplementary material available at 10.1007/s00787-023-02166-z.

## Introduction

Affective dysregulation (AD) is described as a transdiagnostic phenomenon and is characterized by excessive reactivity to emotional stimuli [10]. Children with AD often react to negative events in a particularly irritable way and with severe outbursts of temper, anger, and unpredictable mood swings [23]. This behavior is marked by an elevated use of maladaptive emotion regulation strategies [22]. Accordingly, children with AD exhibit low frustration tolerance and tend to react aggressively when an anticipated reward is withheld [27]. The susceptibility to anger arousal might be explained by cognitive inflexibility in children who show high irritability [34]. While some authors consider AD and irritability as the same construct or as highly similar constructs (e.g., [23, 30], our definition of AD is broader: Whereas irritability comprises only one affective component of AD—proneness to anger [34]—AD additionally comprises emotional reactions other than anger, such as anxiety or sadness, or even positive emotions (such as exuberance). As such a broad construct, AD fits well with the concept of the Research Domain Criteria (RDoC) initiative of the National Institute of Mental Health, which highlights a dimensional and transdiagnostic approach to researching mental disorders [16]. In the RDoC, AD is classified within the construct of frustrative non-reward in the domain of negative emotionality [27].

Since AD does not represent a specific diagnosis but rather a transdiagnostic entity, there have been efforts to operationalize AD in a specific diagnosis: The fifth revision of the Diagnostic and Statistical Manual of Mental Disorders (DSM-5) introduced so-called disruptive mood dysregulation disorder (DMDD) as a new diagnostic entity for severely impaired children with chronic irritability and intense temper outbursts [2]. The recently published eleventh revision of the International Classification of Diseases (ICD-11) introduced chronic irritability and anger as a specifier for the diagnosis of oppositional defiant disorder (ODD) to differentiate children with and without chronic irritability and anger [37].

Depending on the conceptualization of AD, previous studies found prevalence rates of 0.8–9% in school-aged children and adolescents [6, 24]. Children and adolescents with AD suffer from high levels of impairment [6]. Although AD tends to decrease with age, symptoms persist into adolescence in a significant number of children [24]. Given its transdiagnostic nature, AD is found across various mental disorders in childhood and adolescence [10]. Externalizing disorders seem to be especially common in school-aged children and adolescents with AD, the most frequent being ODD, followed by attention-deficit/hyperactivity disorder (ADHD) and conduct disorder (CD; [3, 6]. However, AD is also linked to internalizing disorders in school-aged children and adolescents, most commonly depression, followed by anxiety [6]. In view of the stability of AD, the resulting impairment, and its association with other mental disorders, a reliable and valid measure is needed to adequately identify children at risk.

Currently, the number of assessment tools to measure AD is rather limited. There are some measures assessing certain aspects of AD, such as emotion regulation (Emotion Regulation Checklist; [31], anger (PROMIS Anger Scale [17], or irritability (Affective Reactivity Index [32]. Furthermore, two broadband questionnaires assess the so-called dysregulation profile: the Child Behavior Checklist—Dysregulation Profile (CBCL-DP [1], and the Strengths and Difficulties Questionnaire—Dysregulation Profile (SDQ-DP; [7, 14]. The dysregulation profile is defined as the co-existence of anxious/depressive, attention, and aggressive problems [1]. For both questionnaires, specific subscales/items are combined to form the profile [1, 7, 14]. Lastly, the questionnaires and interviews on disruptive behaviors from the Diagnostic System for Mental Disorders in children and adolescents according to ICD-10 and DSM-5 (in German language: Diagnostik-System für Psychische Störungen, DISYPS-III; [9] also include a DMDD subscale in its third version. These measures, though helpful, do not capture the full picture of AD [36].

The newly developed Diagnostic Tool for Affective Dysregulation in Children (DADYS; [10, 12, 18, 28] might be suitable to fill this gap, as it focuses on the broader conceptualization of AD comprising all of the stated aspects of AD by merging the different operationalizations into one tool. To integrate different perspectives, it comprises parent, self-, and clinical ratings. Furthermore, it includes a parent-rated screening questionnaire—the DADYS-Screen—which might be particularly appropriate to identify children at risk of AD [28]. The DADYS-Screen assesses symptoms of irritability, impulsivity, temper outbursts, anger, and mood swings in 8–12 year-olds. The DADYS measures were developed based on existing measures of aspects of AD (Global Index of the Conners’ Rating Scale, [5],SDQ, [7], DISYPS-III, [9], PROMIS Anger Scale, [17],Emotion Regulation Checklist, [31],Affective Reactivity Index, [32]. Item selection and evaluation for the DADYS-Screen was conducted following a mixed-methods approach, including a Delphi rating of experts, focus groups with experts and parents, and psychometric analyses based on methods from classical test theory and item response theory (see [28] for detailed information on item compilation and selection). The first evaluation of the DADYS-Screen by Otto et al. [28] indicated excellent internal consistency, high content validity, and mainly good psychometric properties and scale characteristics, including a good fit to a one-factorial model. However, the construct and criterion validity have not yet been comprehensively evaluated, and there is no cut-off considering its sensitivity and specificity in relation to comprehensive assessments of AD.

Therefore, in the current study, we analyzed a screened sample of children with and without AD. First, we aimed to demonstrate the criterion validity of the DADYS-Screen by analyzing the concordance with established comprehensive measures of AD (concurrent validity). We hypothesized that the DADYS-Screen would show

- Large to very large correlations with comprehensive measures of AD symptoms;
- An area under the curve (AUC) that is at least acceptable in receiver operating characteristic (ROC) analyses when compared to comprehensive measures of AD.

Second, we aimed to demonstrate the construct validity of the DADYS-Screen by analyzing the concordance with established measures of related constructs (convergent and divergent validity) and the ability to differentiate between groups (discriminant validity). We hypothesized that the DADYS-Screen would show.

c) Moderate to large correlations with measures of emotion regulation strategies as potential maintaining factors of AD;
d) Moderate to large correlations with measures of externalizing symptoms due to the high conceptual overlap;
e) Small to moderate correlations with measures of internalizing symptoms due to the moderate conceptual overlap;
f) Moderate to large correlations with measures of health-related quality of life (HRQoL) due to the high levels of impairment in children with AD;
g) Higher scores for children with diagnoses of DMDD, ODD/CD, ADHD, or depression compared to children without these diagnoses.

Finally, we sought to determine a cut-off for the DADYS-Screen based on the ROC analyses.

## Methods

### Participants

The participants of the present study were part of a larger study on Affective Dysregulation on Optimizing Prevention and Treatment (ADOPT), which aimed at developing assessment tools for diagnosing AD, analyzing the epidemiology, and investigating the efficacy of treatment approaches for children with AD [10]. Within the subproject ADOPT Epidemiology, a large community sample (*n* = 9759) was recruited in four German cities through residents’ registration offices (for detailed information, see [10, 28].

After the initial screening with the DADYS-Screen, all families within the highest 10% raw scores on the DADYS-Screen (sample_highAD_; *n* = 287) were invited to participate in a comprehensive assessment including clinical, parent, and child ratings. Participating families were subsequently randomized to receive either treatment as usual or an AD-specific psychotherapeutic treatment. The cut-off of 10% was chosen as an approximation to epidemiological studies, which found prevalence rates of up to 9% [24]. For the low AD comparison group, a random sample of families within the lowest 10% raw scores (sample_lowAD_, *n* = 184) was invited to participate in the same comprehensive assessment including clinical, parent, and child ratings. To be able to display the full range of AD, we additionally employed comprehensive parent-rated questionnaires in a randomly drawn sample of families within the middle 80% of the raw score distribution (sample_moderateAD_; *n* = 643). Thus, the total sample for the current study comprised 1,114 families. We chose an age range of 8–12 years for the children since the focus of the study was on AD in childhood and we wished to include children’s self-ratings for several questionnaires. Additional inclusion criteria for the comprehensive assessment were IQ above 80, no current behavioral therapy focusing on AD, and no autism spectrum disorder. We did not exclude children with comorbid disorders such as ODD, CD, ADHD, depression, or anxiety as we wished to analyze potential symptom overlaps. The assessment was completed either online via the REDcap electronic data capture tool, hosted at the Clinical Trials Centre Cologne, or offline in paper-and-pencil format. The average time between screening and comprehensive assessment was 15.73 weeks (*SD* = 11.46). Randomization to the treatment condition for the sample_highAD_ was conducted after the completion of the comprehensive assessment.

### Measures

#### DADYS

The DADYS [12, 18, 28] is a comprehensive assessment tool including the screening questionnaire (DADYS-Screen, 12 items), a diagnostic interview for parents (DADYS-PI, 13 items) and children (DADYS-CI, 10 items), and a questionnaire for parents (DADYS-PQ, 36 items) and children (DADYS-CQ, 26 items). As mentioned above, the DADYS comprises symptoms of irritability, impulsivity, temper outbursts, anger, and mood swings (e.g., “is easily annoyed by others”, “exhibits wide mood swings”). Items are rated on a 4-point Likert scale ranging from 0 (not present) to 3 (very strong). For each questionnaire/interview, the mean item score was calculated. In the current sample, the internal consistency of each questionnaire/interview was good to excellent (86 ≤ *α* ≤ 0.96). Additionally, DMDD diagnosis was evaluated based on the DADYS-PI as 0 (no) and 1 (yes).

#### CBCL-DP

As a second measure of AD, we included the Dysregulation Profile [1] of the Child Behavior Checklist in its German version (CBCL/6-18R; [11]. The items of the subscales anxious/depressed (13 items), attention problems (10 items,) and aggressive behavior (18 items) were rated by parents on a 3-point Likert scale ranging from 0 (not true) to 2 (very true or often true). We calculated the mean item score for each subscale. For the CBCL-DP scale, we subsequently summed the scores, resulting in a range from 0 to 6, weighing each scale by the number of items [25]. In the current sample, we analyzed the anxious/depressed subscale and the CBCL-DP scale, which both demonstrated good to excellent internal consistency (*α* = 0.80-0.94).

#### FRUST

We assessed emotion regulation strategies using the Questionnaire for the Regulation of Frustration in children (FRUST; [13], Junghänel [19]). The FRUST comprises the subscales adaptive and maladaptive emotion regulation strategies in the parent (adaptive: 10 items, maladaptive: 4 items) and child rating (adaptive: 33 items, maladaptive: 7 items). Adaptive strategies include, e.g., problem-solving or social support while maladaptive strategies include, e.g., rumination or avoidance. Items were rated on a 5-point Likert scale ranging from 0 (hardly ever) to 4 (almost always). The mean item score of each subscale was calculated. In the current sample, the internal consistency of the subscales was sufficient to excellent (0.78 ≤ *α* ≤ 0.94).

#### DISYPS-III

Child internalizing and externalizing symptoms were assessed using the DISYPS-III [9]. We used the therapist-rated diagnostic screening checklist for internalizing (19 items) and externalizing symptoms (9 items) based on a parent interview, the parent and child-rated symptom checklists for ADHD (20 items) and disruptive disorders—including DMDD, ODD, and CD—(28 items), and the parent-rated symptom checklist post-traumatic stress disorder (PTSD; 19 items). Items were rated on a 4-point Likert scale ranging from 0 (age-typical) to 3 (very strong). The mean item score across all items was calculated per checklist, with the exception of the checklist for disruptive disorders, where we calculated subscales for ODD, and CD. Note that the PTSD scale was only assessed if the child had experienced at least one potentially traumatizing event (*n* = 588). In the current sample, internal consistency was good to excellent (0.79 ≤ *α* ≤ 0.94), with the exception of the CD scale (*α* = 0.61) due to the diverse behaviors assessed in this scale.

Additionally, we evaluated diagnoses of ADHD, disruptive disorders (ODD and/or CD), and depression as 0 (no) and 1 (yes) based on the DISYPS parent interviews. If parents reported symptoms of ADHD, disruptive disorders, or depression on the screening checklist, the respective comprehensive checklist from the DISYPS-III was employed to confirm the diagnosis.

#### KIDSCREEN

HRQoL was assessed using the KIDSCREEN questionnaire (The KIDSCREEN Group Europe, 2006), which assesses subjective health and well-being in children and adolescents. We used the child-rated KIDSCREEN-10 Index (10 items) and the parent-rated short version KIDSCREEN-27 (27 items). Items were rated on a 5-point Likert scale ranging from 1 (never/not at all) to 5 (always/very strong). The mean item score was calculated. In the current sample, internal consistency was good to excellent (0.81 ≤ *α* ≤ 0.91).

### Data analysis

Statistical analyses were performed using SPSS 20 (IBM Corp, 2011). Missing data were imputed using expectation maximization (EM) if at least 70% of the items per scale were available. Items of each respective scale were used for imputation.

Differences in sample characteristics between the subsamples were examined using *χ*^2^ tests for categorical variables and Kruskal–Wallis tests for continuous variables. As measures of effect size, we used Cramer’s V for *χ*^2^ tests (0.10 ≤ *ϕ*_c_ < 0.30 small, 0.30 ≤ *ϕ*_c_ < 0.50 moderate, 0.50 ≤ *ϕ*_c_ large) and Pearson correlations for Kruskal–Wallis tests (0.10 ≤ *r* < 0.30 small, 0.30 ≤ *r* < 0.50 moderate, 0.50 ≤ *r* large; [4].

For the correlation analyses (concurrent, convergent, and divergent validity), we calculated partial rank correlations controlling for age and gender between the DADYS-Screen and comprehensive measures of AD, emotion regulation strategies, externalizing and internalizing symptoms, and measures of HRQoL. Note that we report the correlations of the DADYS-Screen with the DADYS-PQ both for the total scale and for a reduced scale excluding items which were also part of the DADYS-Screen. To avoid item overlap between the validators, we did not calculate the DISYPS-III DMDD subscale due to item overlap with the DADYS-PQ/-CQ. Furthermore, we excluded three items of the ODD subscale in our analyses, which were part of the DMDD subscale and the DADYS-PQ/-CQ. As measures of effect size, the correlation coefficients were interpreted as mentioned above [4]. We classified correlations accounting for at least 50% of the variance (*r* > 0.70) as very large. Additionally, we compared correlation coefficients of the different conceptualizations of AD, externalizing symptoms, and internalizing symptoms according to Meng et al. [26] if at least two measures for the same rater in the same (sub)sample were present.

The AUC (concurrent validity) and the optimal cut-off of the DADYS-Screen were determined using ROC analyses. The ROC curve displays the relation between sensitivity and 1-specificity values. We compared the DADYS-Screen with two measures of the DADYS-PI: a) total score cut-off: total score of at least 1 (yes/no) and (b) DMDD: diagnosis (yes/no). For the total score cut-off, each item must be fulfilled at least mildly. This cut-off is comparable to that used in the Multimodal Treatment Study of Children with ADHD (MTA) study [35]. To quantify the discriminatory power of the DADYS-Screen, we analyzed the AUC for both (a) and (b) [20]. The AUC score ranges from 0.50 (at random) to 1 (perfect). We used the following interpretations of AUC scores: 0.50 ≤ AUC < 0.70 poor, 0.70 ≤ AUC < 0.80 acceptable, 0.80 ≤ AUC < 0.90 excellent, 0.90 ≤ AUC outstanding [15]. To determine the optimal cut-off for the DADYS-Screen, we employed the Youden Index [38], which aims to maximize the distance between the line of equal sensitivity and specificity (diagonal line) and the point farthest from this line [20]. Thus, the highest score demonstrates the best cut-off. Based on this cut-off, we analyzed sensitivity and specificity.

Additionally, we tested differences between the DADYS-Screen scores of children with and without a diagnosis of DMDD, disruptive disorders, ADHD, depression, or any of these diagnoses using Mann–Whitney *U* tests (discriminant validity). To account for differences in age and gender, we implemented a case–control matching for each child with a diagnosis. As a measure of effect size, we used Pearson correlations.

## Results

### Participant characteristics

Participant characteristics are displayed in Table 1. There were no differences between subsamples regarding the migration background of the child or regarding family characteristics. As expected, a small to large effect for parent-rated AD on the DADYS-Screen emerged, with the highest scores in sample_highAD_ and the lowest in sample_lowAD_. Moreover, there were significantly more boys in sample_highAD_ compared to the other two samples (small effect). A small to moderate effect of child age was found, with the youngest children in sample_moderateAD_ and the oldest in sample_lowAD._

**Table 1:** Participant characteristics for subsamples

	Range	Total sample	sample_lowAD_	sample_moderateAD_	sample_highAD_	test statistic	effect
		(*n* = 1114)	(*n* = 184)	(*n* = 643)	(*n* = 287)		
Child variables							
Gender (male): %	1, 2	52.8%	46.2%	49.9%	63.4%	*χ*^2^(2) = 18.33,* p* < 0.001	*ϕ*_c_ = 0.13
Age (years): *M* (*SD*)	8–12	10.20 (1.49)	10.93 (1.35)	9.85 (1.51)	10.52 (1.28)	*χ*^2^(2) = 99.49,* p* < 0.001	*r* = 0.13–0.31
Migration background^a^: %	0, 1	21.4%	21.2%	21.8%	20.6%	*χ*^2^(2) = 0.18, *p* = 0.915	–
DADYS-Screen^b^: *M* (*SD*)	0–3	0.83 (0.75)	0.22 (0.15)	0.50 (0.36)	1.95 (0.36)	*χ*^2^(2) = 691.76,* p* < 0.001	*r* = 0.25–1.07
Family variables							
Single parent status: %	0, 1	10.7%	9.3%	10.3%	12.5%	*χ*^2^(2) = 1.51, *p* = 0.470	–
Socioeconomic status^c^: *M* (*SD*)	1–7	6.21 (1.23)	6.34 (1.17)	6.18 (1.25)	6.19 (1.22)	*χ*^2^(2) = 3.40,* p* = 0.182	–

Test statistics are based on *χ*^2^-tests for categorical variables and Kruskal–Wallis tests for continuous variables

*ϕ*_*c*_ effect size Cramer’s V for *χ*^2^-tests, *r* effect size Pearson correlation for Kruskal–Wallis tests, *M* mean, *SD* standard deviation, *DADYS-Screen* Diagnostic Tool for Affective Dysregulation in Children-Screening Questionnaire

^a^Child or at least one parent born outside of Germany

^b^Caregiver rating

^c^Value is based on the average national income obtained with the highest education and occupational qualification in the family [21]

### Concurrent validity

The results of all correlation analyses are presented in Table S1 of the supplement. As expected, the DADYS-Screen demonstrated very strong correlations with comprehensive measures of AD. Correlations were very large for parent questionnaires and clinical interviews (0.70 ≤ *r* ≤ 0.83) and large for child questionnaires (0.64 ≤ *r* ≤ 0.67). The association of the DADYS-PQ with the DADYS-Screen was stronger than the association of the CBCL-DP with the DADYS-Screen (*z* = 10.78, *p* < 0.001)—even if DADYS-Screen items were excluded from the DADYS-PQ (*z* = 4.80, *p* < 0.001).

**Fig. 1:**
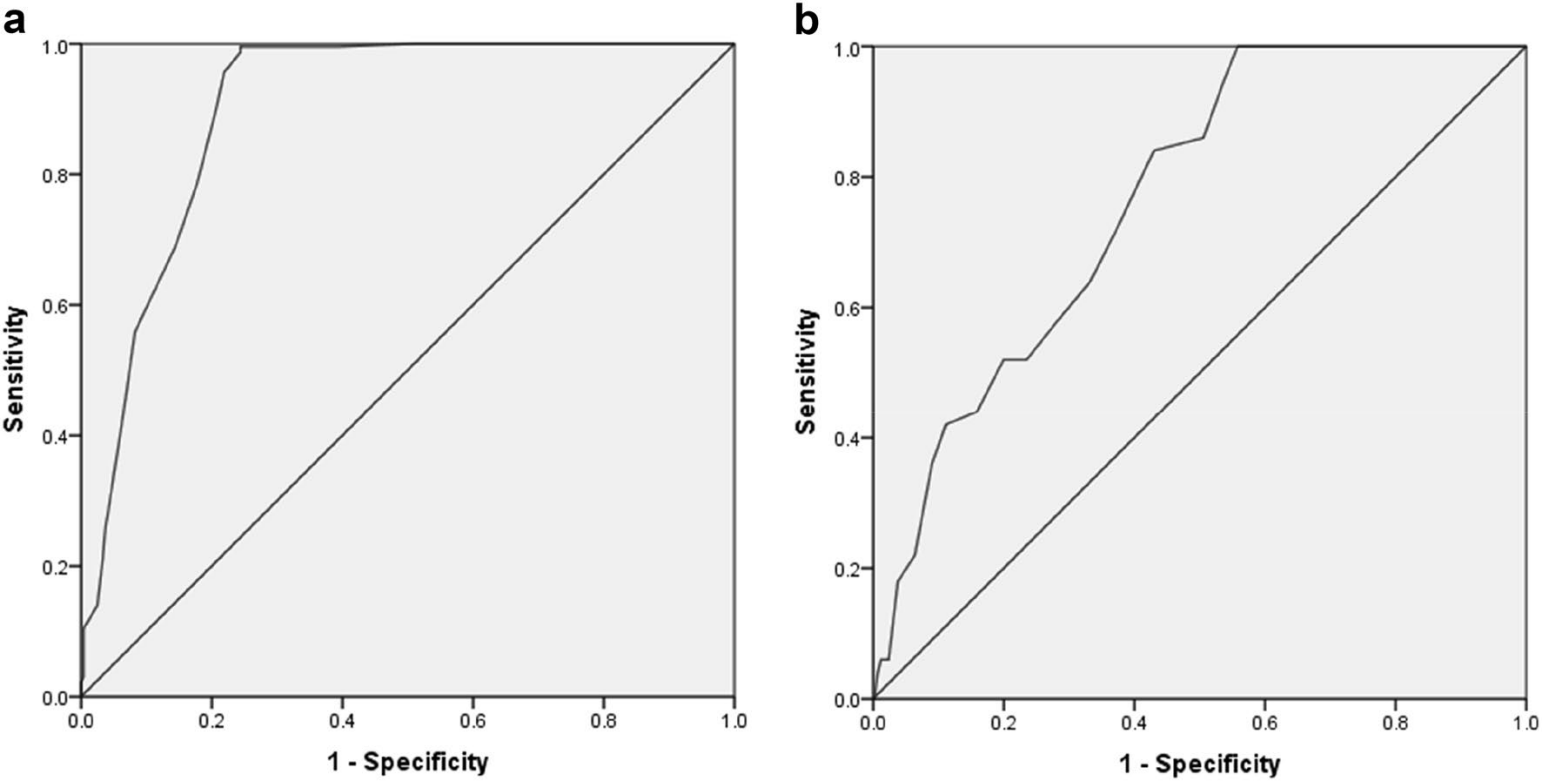
**a** ROC curve of DADYS-Screen for DADYS parent interview total score cut-off. **b** ROC curve of DADYS-Screen for DADYS parent interview DMDD diagnosis

ROC analyses revealed outstanding diagnostic accuracy of the DADYS-Screen for the DADYS-PI total score cut-off (AUC = 0.90; 95% CI = 0.87-0.93) and acceptable diagnostic accuracy for the DMDD diagnosis (AUC = 0.77; 95% CI = 0.71–0.82). Figure 1 shows the ROC curve for the DADYS-PI total score cut-off (a) and the DMDD diagnosis (b).

### Convergent and divergent validity

For emotion regulation strategies, large correlations—positive for maladaptive, negative for adaptive strategies—emerged for parent-rated questionnaires (0.56 ≤ *r* ≤ 0.62) and moderate correlations emerged for child-rated questionnaires (0.30 ≤ *r* ≤ 0.40).

As expected, the DADYS-Screen correlated more strongly with measures of externalizing compared to internalizing symptoms assessed in clinical ratings (*z* = 6.77, *p* < 0.001). In detail, we found very large correlations with externalizing symptoms for clinical ratings (*r* = 0.71), large correlations for parent ratings (0.52 ≤ *r* ≤ 0.67), and moderate to large correlations for child ratings (0.39 ≤ *r* = 0.51). There were significant differences between the correlations of ODD, ADHD, and CD symptoms in parent ratings (*z* = 2.63–7.65, *p* ≤ 0.008), with the largest correlations for ODD symptoms and the smallest correlations for CD symptoms. In child rating, we also found stronger associations of the DADYS-Screen with ODD and ADHD than with CD symptoms (*z* = 2.57–3.81, *p* ≤ 0.010). However, the difference between the correlations of ODD and ADHD did not reach statistical significance (*z* = 1.09, *p* = 0.277).

For measures of internalizing symptoms, we detected large correlations for clinical ratings (*r* = 0.50) and moderate correlations for parent ratings (0.39 ≤ *r* ≤ 0.46). Compared to PTSD symptoms, we found stronger correlations of the DADYS-Screen with anxious/depressed symptoms (*z* = 3.07, *p* = 0.002).

The negative correlations of the DADYS-Screen with measures of HRQoL were large for parent ratings (*r* = − 0.55) and moderate for child ratings (*r* = − 0.40).

### Discriminant validity

The results of the analyses for discriminant validity are presented in Table 2. Due to the low number of diagnoses of depression in our sample (*n* = 12), we excluded this variable from the analyses. On the DADYS-Screen, children with diagnoses of DMDD, disruptive disorders, ADHD, or any diagnosis scored higher than children without these diagnoses (moderate to large effects). The greatest effects between diagnoses were found for disruptive disorders and any diagnoses, with large effects.

**Table 2:** Analyses of discriminant validity of the DADYS-Screen

Diagnosis		*n*	DADYS-screen		Test statistic	Effect
			*M*	*SD*		
DMDD	No	50	1.21	0.88	*U* = 602.50; *p* < 0.001	*r* = 0.45
	Yes	50	2.05	0.42		
Disruptive disorder	No	111	0.99	0.89	*U* = 2463.00; *p* < 0.001	*r* = 0.52
	Yes	111	2.02	0.37		
ADHD	No	74	1.07	0.88	*U* = 1387.00; *p* < 0.001	*r* = 0.43
	Yes	74	1.91	0.38		
Any disorder	No	156	0.91	0.85	*U* = 4339.50; *p* < 0.001	*r* = 0.56
	Yes	156	1.96	0.38		

Test statistics are based on Mann–Whitney *U* tests

*M* mean, *SD* standard deviation, *r* effect size Pearson correlation, *DADYS-Screen* Diagnostic Tool for Affective Dysregulation in Children—Screening Questionnaire, *DMDD* disruptive mood dysregulation disorder, *ADHD* attention-deficit/hyperactivity disorder

^***^p < 0.001

### Determination of cut-off

Sensitivity and specificity for all potential cut-off points can be found in Table S2 for the DADYS-PI total score cut-off and Table S3 for the DMDD diagnosis in the supplement. For the DADYS-PI total score cut-off, the Youden Index indicated a cut-off of 0.88 as optimal—with a sensitivity of 99.6% and a specificity of 75.5%. For the DMDD diagnosis, the Youden Index indicated a cut-off of 1.38 as optimal with a sensitivity of 100.0% and a specificity of 44.2%.

## Discussion

The present study analyzed associations of the parent-rated DADYS-Screen with comprehensive measures of AD and related mental disorders for validation in a community sample of children with and without AD. We demonstrated criterion validity of the DADYS-Screen through (very) large correlations with more comprehensive measures of AD, an acceptable diagnostic accuracy in ROC analyses with the DMDD diagnosis, and an outstanding diagnostic accuracy with the DADYS-PI total score cut-off. Furthermore, construct validity was demonstrated through meaningful correlations with measures of externalizing and internalizing symptoms, emotion regulation strategies, and HRQoL, and through significantly higher DADYS-Screen scores in children with mental disorders linked to AD. Lastly, we provided an index for an optimal cut-off for the DADYS-Screen.

As hypothesized, the DADYS-Screen showed the strongest associations with comprehensive measures of AD. The largest correlation was demonstrated by the DADYS-PQ—even when excluding overlapping items. Additionally, we found an outstanding diagnostic accuracy of the DADYS-Screen with the DADYS-PI. These results might be explained by the fact that the DADYS is the only tool focusing on the broad conceptualization of AD. In contrast, the CBCL-DP focuses on severely dysregulated children with anxious/depressive, attention, and aggressive problems [14]. Thus, it emphasizes emotional aspects more strongly than the DADYS, which might explain the slightly lower correlation with the DADYS-Screen compared to the DADYS-PQ. In our study, we chose the continuous score of the CBCL-DP to display the full range of AD [25]. Another common operationalization is a cut-off for each scale (e.g., [8]. Our approach might have led to slightly higher correlations compared to the cut-off score.

We also found an association of the DADYS-Screen with the diagnosis of DMDD. In detail, we found moderately higher scores on the DADYS-Screen in children with a DMDD diagnosis and we found an acceptable diagnostic accuracy in the ROC analyses. Although DMDD focuses on chronic irritability and temper outbursts [2], these symptoms are also aspects of the broad conceptualization of AD. However, these aspects may reflect its most extreme expression and may focus more strongly on the emotional aspects of anger. The different foci might also explain the lower diagnostic accuracy of the DADYS-Screen for the DMDD diagnosis compared to the outstanding diagnostic accuracy of the broader DADYS-PI.

As expected, the DADYS-Screen showed a positive correlation for maladaptive emotion regulation strategies and a negative correlation for adaptive strategies. Legenbauer et al. [22] also reported this pattern of findings in adolescent patients. Accordingly, the use of emotion regulation strategies might be an underlying mechanism of AD [22]. For HRQoL, we found a negative correlation, as expected based on previous literature on impairment due to AD [6]. The moderate to large effects are comparable to previous studies (e.g., [29]. It would be especially interesting to analyze emotion regulation strategies and HRQoL in a longitudinal study, as there might be a causal impact of emotion regulation strategies on AD, which in turn might decrease HRQoL.

In line with our hypothesis, the DADYS-Screen showed higher correlations for externalizing compared to internalizing symptoms and a moderate to strong discriminative effect. The strongest correlations emerged for measures of ODD (and ADHD for child ratings)—even though we excluded overlapping items of the ODD scales—and the weakest correlations emerged for measures of CD. This pattern largely corresponds to previous literature [3, 6]. The especially strong connection between AD and ODD might be attributable to their symptom overlap [3]. However, AD might also be a risk factor for the development of ODD. Stringaris et al. [33] found that ODD was predicted by early emotionality and activity. While early emotionality predicted ODD with internalizing disorders more strongly, early activity predicted ODD with ADHD and conduct problems more strongly. The authors considered early emotionality as an antecedent of the irritability dimension in ODD. Thus, early emotionality might be similar to the concept of AD, which would explain the stronger associations with ODD compared to ADHD and CD.

For internalizing symptoms, we found a moderate to large overlap with the DADYS-Screen. In accordance with previous literature [6], which reported a stronger association of AD with depression than with anxiety, we found smaller correlations with PTSD compared to a combined measure of anxiety and depression. Based on our hypothesis, we expected small to moderate overlap with measures of internalizing symptoms. Thus, the large association in clinical ratings seemed surprising, particularly since our sample did not comprise many children with internalizing disorders. However, as mentioned above, the association with internalizing symptoms was still lower than the association with externalizing symptoms—in line with previous research. Future studies might analyze the associations of AD with depression and anxiety in more detail in a sample comprising more children with anxiety disorders and depression.

Finally, for the broader concept of AD, we found good sensitivity and specificity for the indicated optimal cut-off, with a higher score for sensitivity. If a child surpasses this cut-off, we recommend more comprehensive diagnostics for AD and related disorders to narrow down individual problems and eliminate the small possibility of a false-positive screening. For the DMDD diagnosis, we found good sensitivity but unacceptable specificity for the suggested optimal cut-off. This finding further strengthens our argument that differences in the conceptualizations of DMDD and AD are especially apparent when applying dichotomous measures. Therefore, we do not recommend employing the DADYS-Screen as a screening questionnaire for the DMDD diagnosis but do recommend it for the broader concept of AD.

Limitations of the present study include the age range of our sample, meaning that the findings cannot be generalized to individuals younger than 8 and older than 12 years. Regarding the early detection of AD, future research may wish to investigate the feasibility and validity of the DADYS-Screen for use in younger children. Additionally, a self-report version of the DADYS-Screen would be beneficial for wider use to screen children with AD symptoms. Moreover, parents with lower and medium socioeconomic status were underrepresented in our sample, potentially suggesting a relation between willingness to participate and socioeconomic status. Lastly, we were not able to assess all measures in all raters. As mentioned above, clinical and child report was only assessed in the sample of children with low and high AD. While we only found small differences in correlations in parent-rated measures for the total versus the extreme sample (*r*_mean difference_ = 0.02), we cannot rule out an overestimation of the effects due to the restricted variance for child and clinical ratings. Strengths of the study include the large sample size, the investigation of children with and without AD (and with ADHD, disruptive disorders, and depression), and the inclusion of different perspectives (parent, child, and clinical ratings).

In conclusion, this study contributes to the understanding and assessment of children with AD. We demonstrated that all applied measures of AD assess some form of AD, but with different conceptual foci. Measures of externalizing symptoms showed a stronger overlap than measures of internalizing symptoms. We also found associations with emotion regulation strategies and HRQoL. Finally, we demonstrated that the DADYS-Screen can adequately identify children at risk of AD from a screened community sample, and provided an optimal cut-off. Considering our results and those of Otto et al. [28], we conclude that the DADYS-Screen appears to be a reliable and valid measure to identify school-aged children at risk of AD. Such accurate assessment measures enable a comprehensive understanding and adequate tailoring of treatment methods to the individual problems of each child.

### Supplementary Information

Below is the link to the electronic supplementary material.

Supplementary file1 (DOCX 55 KB)

## Data Availability

The dataset can be obtained from the corresponding author after publication of the main outcome analyses on treatment effects.
