# Supplementary material for: Screening for affective dysregulation in school-aged children: relationship with comprehensive measures of affective dysregulation and related mental disorders
Source: Eur Child Adolesc Psychiatry. 2023 Feb 17;33(2):381–90. doi: 10.1007/s00787-023-02166-z (PMC10869411; doi:10.1007/s00787-023-02166-z)
Supplement: Supplementary file 1 — Supplementary file1 (DOCX 55 KB) [file 787_2023_2166_MOESM1_ESM.docx]

**Supplement**

**Screening for Affective Dysregulation in School-Aged Children: Relationship with Comprehensive Measures of Affective Dysregulation and Related Mental Disorders**

**Table S1**

*Correlation analyses of the DADYS-Screen*

| **measure** | **rater** | **sample** | ***r^a^*** |
| --- | --- | --- | --- |
| Correlations with other measures of affective dysregulation | | | |
| DADYS-PQ: total scale | parent | total | .83 |
| DADYS-PQ: total scale without DADYS-Screen items | parent | total | .77 |
| CBCL: dysregulation profile | parent | total | .71 |
| DADYS-PI: total scale | clinician | low, high | .75 |
| DADYS-CI: total scale | clinician | low, high | .70 |
| DADYS-CQ: total scale | child | low, high | .67 |
| Correlations with measures of emotion regulation strategies^b^ | | | |
| Maladaptive strategies | parent | total | .62 |
| Adaptive strategies | parent | total | -.56 |
| Maladaptive strategies | child | low, high | .40 |
| Adaptive strategies | child | low, high | -.30 |
| Correlations with measures of externalizing symptoms^c^ | | | |
| ODD scale without DMDD items | parent | total | .67 |
| CD scale | parent | total | .52 |
| ADHD scale | parent | total | .58 |
| Externalizing symptoms scale | clinician | low, high | .71 |
| ODD scale without DMDD items | child | low, high | .51 |
| CD scale | child | low, high | .39 |
| ADHD scale | child | low, high | .47 |

| Correlations with measures of internalizing symptoms^c^ | | | |
| --- | --- | --- | --- |
| CBCL: anxious/depressed scale | parent | total | .46 |
| DISYPS-III: PTSD scale | parent | total | .39 |
| DISYPS-III: internalizing symptoms scale | clinician | low, high | .50 |
| Correlations with measures of health-related quality of life^d^ | | | |
| Total scale | parent | total | -.55 |
| Total scale | child | low, high | -.40 |

Note. DADYS = Diagnostic Tool for Affective Dysregulation in Children. DISYPS-III = Diagnostic System for Mental Disorders in children and adolescents. CBCL = Child Behavior Checklist. PQ = parent questionnaire. CQ = child questionnaire. PI = parent interview. CI = child interview. DMDD = disruptive mood dysregulation disorder. ODD = oppositional defiant disorder. CD = conduct disorder. ADHD = attention-deficit/hyperactivity disorder. PTSD = post-traumatic stress disorder.

^a^all *p* < .001.

^b^assessed with the Questionnaire for the Regulation of Frustration in children (FRUST).

^c^assessed with the DISYPS-III.

^d^assessed with the KIDSCREEN.

**Table S2**

*Potential cut-off points with sensitivity, specificity, and Youden Index of the DADYS-Screen for DADYS parent interview total score cut-off*

| Cut-off | sensitivity | specificity | Youden |
| --- | --- | --- | --- |
| 0.04 | 1.000 | 0.128 | 0.128 |
| 0.13 | 1.000 | 0.219 | 0.219 |
| 0.21 | 1.000 | 0.38 | 0.380 |
| 0.29 | 1.000 | 0.488 | 0.488 |
| 0.38 | 0.996 | 0.607 | 0.603 |
| 0.46 | 0.996 | 0.715 | 0.711 |
| **0.88** | **0.996** | **0.756** | **0.752** |
| 1.29 | 0.991 | 0.756 | 0.748 |
| 1.38 | 0.987 | 0.756 | 0.743 |
| 1.46 | 0.956 | 0.781 | 0.737 |
| 1.54 | 0.900 | 0.793 | 0.693 |
| 1.63 | 0.786 | 0.822 | 0.608 |
| 1.71 | 0.690 | 0.855 | 0.545 |
| 1.79 | 0.629 | 0.884 | 0.513 |
| 1.88 | 0.559 | 0.917 | 0.476 |
| 1.96 | 0.472 | 0.93 | 0.401 |
| 2.04 | 0.415 | 0.938 | 0.353 |
| 2.13 | 0.336 | 0.95 | 0.287 |
| 2.21 | 0.258 | 0.963 | 0.221 |
| 2.29 | 0.210 | 0.967 | 0.177 |
| 2.38 | 0.140 | 0.975 | 0.115 |
| 2.46 | 0.105 | 0.996 | 0.101 |
| 2.54 | 0.079 | 0.996 | 0.075 |
| 2.63 | 0.052 | 0.996 | 0.048 |
| 2.71 | 0.035 | 0.996 | 0.031 |
| 2.79 | 0.031 | 0.996 | 0.026 |
| 2.88 | 0.022 | 1 | 0.022 |
| 2.96 | 0.004 | 1 | 0.004 |

**Note:** Optimal cut-off according to Youden Index in bold.

**Table S3**

*Potential cut-off points with sensitivity, specificity, and Youden Index of the DADYS-Screen for DMDD diagnosis*

| cut-off | sensitivity | specificity | Youden |
| --- | --- | --- | --- |
| 0.04 | 1.000 | 0.074 | 0.074 |
| 0.13 | 1.000 | 0.126 | 0.126 |
| 0.21 | 1.000 | 0.219 | 0.219 |
| 0.29 | 1.000 | 0.28 | 0.280 |
| 0.38 | 1.000 | 0.352 | 0.352 |
| 0.46 | 1.000 | 0.413 | 0.413 |
| 0.88 | 1.000 | 0.437 | 0.437 |
| 1.29 | 1.000 | 0.439 | 0.439 |
| **1.38** | **1.000** | **0.442** | **0.442** |
| 1.46 | 0.940 | 0.466 | 0.406 |
| 1.54 | 0.860 | 0.494 | 0.354 |
| 1.63 | 0.840 | 0.57 | 0.410 |
| 1.71 | 0.720 | 0.627 | 0.347 |
| 1.79 | 0.640 | 0.667 | 0.308 |
| 1.88 | 0.580 | 0.717 | 0.297 |
| 1.96 | 0.520 | 0.765 | 0.285 |
| 2.04 | 0.520 | 0.8 | 0.321 |
| 2.13 | 0.440 | 0.841 | 0.281 |
| 2.21 | 0.420 | 0.888 | 0.308 |
| 2.29 | 0.360 | 0.91 | 0.270 |
| 2.38 | 0.220 | 0.936 | 0.156 |
| 2.46 | 0.180 | 0.962 | 0.142 |
| 2.54 | 0.120 | 0.969 | 0.089 |
| 2.63 | 0.060 | 0.976 | 0.036 |
| 2.71 | 0.060 | 0.986 | 0.046 |
| 2.79 | 0.060 | 0.988 | 0.048 |
| 2.88 | 0.040 | 0.993 | 0.033 |
| 2.96 | 0.000 | 0.998 | -0.002 |

**Note:** Optimal cut-off according to Youden Index in bold.
